# Effect of community attitudes on suicide mortality in South Korea: a nationwide ecological study

**DOI:** 10.3389/fpsyt.2024.1423609

**Published:** 2024-09-16

**Authors:** Minseok Hong, Hyesoo Kim, C. Hyung Keun Park, Hyunju Lee, Sang Jin Rhee, Sooyeon Min, Min Ji Kim, Jeong Hun Yang, Yoojin Song, Kyunghoon Son, Yong Min Ahn

**Affiliations:** ^1^ Department of Psychiatry, Seoul National University College of Medicine, Seoul, Republic of Korea; ^2^ Department of Psychiatry, Asan Medical Center, Seoul, Republic of Korea; ^3^ Department of Neuropsychiatry, Seoul National University Hospital, Seoul, Republic of Korea; ^4^ Department of Clinical Medical Sciences, Seoul National University College of Medicine, Seoul, Republic of Korea; ^5^ Seoul National University Medical Research Center, Institute of Human Behavioral Medicine, Seoul, Republic of Korea

**Keywords:** suicidal ideation, completed suicide, attitudes toward suicide, suicide rate, social stigma, risk factors, general population survey, suicide prevention

## Abstract

**Background:**

Attitudes toward suicide are essential in suicide prevention, as suicide is socio-culturally nuanced. Although the relationship between individual attitudes and suicidal behavior has been extensively studied, the effect of community attitudes—aggregated by region—on suicide mortality remains ambiguous. This study explored the association between community attitudes and real-world suicide mortality.

**Methods:**

Data on attitudes toward suicide from the 2018 Korea National Suicide Survey (N = 1500) and individual mortality data from the MicroData Integrated System were obtained. Confirmatory factor analysis supported a factor structure with three factors: “Permissiveness,” “Unjustified behavior,” and “Readiness to help/Preventability.” Thirty regional units in South Korea aggregated the data for ecological analysis. We used negative binomial models to examine the association at the regional level, and stratified analysis by gender and age group was conducted.

**Results:**

“Permissiveness” was associated with reduced suicide rates in a univariate model (P < 0.001). Adjusting for gender, age, and additional sociodemographics did not alter the association. Additionally, this relationship was observed in males and individuals under 60 years of age after stratification. However, “Unjustified Behavior” and “Readiness to help/Preventability” exhibited no significant association with suicide in any model or stratum.

**Conclusion:**

The observed inverse association between permissive community attitudes and suicide contradicts the findings of previous research that links permissive individual attitudes to increased suicidal behavior. Our findings suggest that attitudes may operate differently at the individual and group levels. Although the cross-sectional design and single-country focus of this study warrant further investigation, our findings indicate that attitudes are significant contextual factors in the process of suicide, which could lead to novel approaches in suicide prevention.

## 1 Introduction

Annually, more than 700,000 people die by suicide (1). South Korea has the highest suicide rate among the Organisation for Economic Co-operation and Development (2). Moreover, suicide is the leading cause of death in Korea for individuals in their teens to thirties (3). Given its prominence as a national issue, Korea has made strenuous efforts over the last decade to reduce the suicide rate. These initiatives included reducing exposure to known means of suicide (4), implementing nationwide suicide prevention education (5), and creating guidelines for suicide reporting in the media (6). Consequently, the suicide rate declined from 2010 to 2016 (7). However, despite ongoing efforts, the suicide rate has not continued to decrease, and alarmingly, youth suicide has risen since then (8). A fresh perspective and approach are required to enhance our understanding and prevention of suicide.

Although mental health issues are a main risk factor for suicide (9–11), suicide is a multifaceted phenomenon that also occurs within a socio-cultural context. Since Durkheim’s classic work on suicidology (12), even contemporary influential theories such as the interpersonal theory (13, 14), the integrated motivational-volitional model (IMV) (15), and the three-step theory (3ST) (16) have highlighted the social dimensions of suicide. Attitudes play a critical role in integrating individual and social perspectives on one’s behavior (17), particularly regarding suicide (18). Evidence indicates that attitudes toward suicide are involved in the social interplay surrounding suicide. Attitude toward suicide is affected by the suicide of a close person and the suicidal intensity (19); it may be involved in suicide clustering among adolescents (20). Therefore, investigating community attitudes, which are aggregated at the regional or group level, is theoretically significant and could offer new insights into suicide prevention.

In recent years, numerous studies have explored the relationship between attitudes toward suicide and suicidal behavior, such as suicidal ideation and attempts. Research from various socio-cultural backgrounds consistently demonstrates that permissive or pro-suicidal attitudes are associated with increased suicidal behavior at the individual level (21–31). A study conducted in Korea, Japan and US found that permissive attitudes were the most significant predictor of the intensity of suicidal ideation across all three countries (18). However, most of these studies have focused on how suicidal behavior changes according to attitudes at the individual level. The potential effect of community attitudes on suicide has been overlooked despite its recognized necessity (25).

Furthermore, the relationship between attitudes toward suicide and actual suicide remains unclear, likely because of difficulties measuring an inner attitude through psychological autopsies. Suicide is a distinguished phenomenon from a suicide attempt. Suicide attempts had an estimated incidence at least 20 times higher than suicides (32), whereas psychological autopsy studies show that more than half of suicides result in death at the first attempt (33). Differences in the method have been observed between suicides and suicide attempts (34), emphasizing the necessity to examine the characteristics of suicide mortality separately.

Despite the pressing need for further investigation, the limited literature on this topic has yielded inconsistent results. Specifically, while Neeleman et al. found that pro-suicidal attitudes toward suicide are associated with increased suicide rates (35), Reynder et al. reported a non-significant association (36). In Sweden, the suicide rate has decreased since the 1980s, whereas permissive attitude ratings have increased (25). Some studies have merely compared two regions (21, 37), resulting in a low level of evidence. Moreover, most of these studies were primarily based in European and North American countries, and research in different socio-cultural contexts is required.

This exploratory study examined the association between attitudes toward suicide and the suicide rate through an ecological analysis, a research methodology investigating relationships between variables at an aggregated level (38). We combined a nationwide survey and statistics, ensuring representativeness, and presented several regression models with varying covariate inputs to accomplish this. To the best of our knowledge, this study is the first to explore the relationship between attitudes and suicide mortality using a nationwide survey and mortality microdata. This group-level investigation will contribute to a deeper understanding of suicide as a biopsychosocial phenomenon. Enhancing our comprehension of the multifaceted nature of suicide may strengthen suicide prevention efforts (39, 40) and provide insight into the stagnant suicide rate in Korea.

## 2 Materials and methods

### 2.1 Participants

The 2018 Korea National Survey on Suicide (KNSS) (41), sponsored by the Ministry of Health and Welfare of Korea, served as the basis for this study. This survey was conducted based on a complex sample design to ensure representativeness. Structured face-to-face interviews were conducted with 1,500 adults aged 19 to 75 between November 21 and December 17, 2018. The interviewers were trained by experts and supported during the survey. Ten households were randomly selected for each district, and an interviewer visited each household and conducted the survey. Following the interview, 30% of the participants underwent phone follow-up for quality control.

The Seoul National University Hospital Institutional Review Board approved and monitored this study (IRB No. 1810-062-979). Interviewers obtained informed consent from participants before commencing the interviews. This study adhered to the Strengthening the Reporting of Observational Studies in Epidemiology reporting guidelines (42) and was conducted in compliance with the Declaration of Helsinki.

### 2.2 Measurements

Demographic characteristics of participants, including age, gender, education, employment, and religion, were collected. Participants were also asked about any previous experience with suicidal ideation.

The Questionnaire on Attitudes Toward Suicide (ATTS) was employed to assess the normative attitudes of participants toward suicide. Developed and validated by Renberg and Jacobsson (25), the ATTS is suitable for large-scale studies and widely used in various countries (43). The Korean version, created through the 2013 KNSS (44), was utilized in the 2018 KNSS. Each item of the ATTS was scored on a Likert scale of 1-5 points, where 1 indicated “disagreement” and 5 indicated “agreement.”

#### 2.2.1 Factor structure of ATTS

Kim et al. previously reported a factor structure of ATTS based on the 2013 and 2018 KNSS (45). In this study, we reevaluated the previous factor structure, as multiple methods for determining the number of factors indicated that a three-factor structure best fits the 2018 KNSS data alone (**Supplementary Figure 1**). Unreliable and semantically redundant factors were removed or combined to create a more parsimonious structure while maintaining the basic framework of the original factor structure. The revised factor structure encompassed 3 factors and 15 items (**Table 1**). Through confirmatory factor analysis (**Supplementary Figure 2**), we assessed the goodness of fit and acquired factor score. The revised factor structure had a good absolute fit and parsimony correction, and the comparative fit was within an acceptable or satisfactory range (46). Acquisition of factor score used Barlett’s method.

**Table 1:** Revised factor structure for the Questionnaire on Attitudes Toward Suicide.

Factor	Number	Items	Mean	SE	Reliability
Permissiveness (PA)	5	Suicide acceptable for incurable disease	2.6	0.041	α = 0.70 ω = 0.78
	16	Situations where suicide is the only solution	2.6	0.044	
	18	Suicide as relief	2.7	0.044	
	20	Consider suicide if incurable disease—self	2.9	0.046	
	34	Right to commit suicide	2.5	0.042	
	36	Get help for suicide if incurable disease—self	3.1	0.040	
Unjustified behavior (UB)	2	Suicide never justifiable	3.9	0.037	α = 0.55 ω = 0.61
	3	Suicide is among the worst for relatives	4.3	0.032	
	19	Youth suicides are particularly puzzling	3.6	0.056	
	27	Express suicide wish without meaning it—self	3.5	0.039	
Readiness to help/ Preventability (RP)	1	Always able to help	3.3	0.052	α = 0.50 ω = 0.57
	9	Duty to restrain suicidal act	3.9	0.036	
	30	Ready to help a suicidal person—self	3.2	0.038	
	33	Suicide talkers are not always completers	3.7	0.036	
	37	Suicide preventable	3.9	0.032	

Items were scored on a Likert scale ranging from 1 to 5, with 1 indicating disagreement and 5 indicating agreement. Reliability coefficients were calculated using Cronbach’s alpha and omega total. English expressions for each item are shortened. SE standard error, α Cronbach’s alpha, ω Omega total.

The factors names, “Permissiveness (PA),” “Unjustified behavior (UB)” and “Readiness to help/Preventability (RP),” were derived from the original factor structure. PA semantically represents permissive and acceptable attitudes toward suicide, considering it a right or option in certain circumstances, such as incurable diseases. UB represents opposition to justifying suicide and regards it as morally reprehensible behavior that should be restrained. Intriguingly, this factor also included items that perceive suicide as puzzling or deceptive. RP represents proactive attitudes toward suicide prevention, characterized by a readiness to help and taking an active role in preventing suicide. Compared to PA, participants in the 2018 KNSS generally demonstrated greater agreement with items in UB and RP, and their standard errors were narrower for most items. The reliability of UB and RP, evaluated using Cronbach’s alpha, is low; however, it surpasses the requisite value of 0.5 for preliminary or exploratory research, as proposed by Nunnally (47).

#### 2.2.2 Suicide mortality

Microdata on suicide mortality were obtained through the MicroData Integrated System (MDIS) provided by Statistics Korea (48). The MDIS provides individual information, including the deceased’s gender, age, address, and cause of death, as registered in the Causes of Death Statistics. Researchers remotely accessed and processed the data stored on the MDIS server, and Statistics Korea examined and approved the anonymization of the processed data when exported. This study included individuals who died between January 1 and December 31, 2018, and whose causes of death corresponded to the X60-X84 (intentional self-harm) codes of the Korean Standard Classification of Diseases 7^th^ edition (49). Consistent with the participants in the KNSS, only death cases involving individuals aged 19 to 75 were included in the analysis.

### 2.3 Statistical analysis

The country was divided into 30 regional units for ecological analysis. Survey strata comprising 80 or more participants were reorganized into clusters of 40 to 70 participants based on geographical proximity, forming regional units (**Supplementary Table 1**). Basic characteristics, ATTS, and microdata on suicide mortality were aggregated by these regional units, utilizing survey weights based on a complex sample design. Comparisons were conducted between regions with high and low suicide rates to demonstrate differences in regional characteristics according to suicide rates. If the Shapiro-Wilk test rejected the normal distribution, Wilcoxon’s test was employed for comparison; otherwise, Welch’s t-test was utilized.

In the preliminary analysis, regional suicide counts exhibited overdispersion, with a mean substantially lower than the variance (386.2 vs. 15924.5). As the negative binomial regression model can be generalized to overdispersed count data in place of Poisson models (50), this model was employed to predict suicide mortality. A univariate model (Model_univ_) and four multivariate models (Model 1 to Model 4) were constructed. In Model 1, adjustments were made for the proportions of females and adults over 60 years. Using the proportion of older adults as a covariate rather than the mean age allowed for consideration of South Korea’s unique characteristics, where elderly suicide is notably prevalent, and its etiology appears to be differentiated (51, 52). In Models 2 and 3, one additional covariate from Model 1 was further adjusted by adding lifetime experience of suicidal ideation (Model 2) and college education (Model 3). In Model 4, a quadratic term of the factor score was added to the adjustments made in Model 1. The dependent variable was the incidence of suicide in each region, with the regional population set as an offset variable. The result of each model was presented as an incidence rate ratio (IRR), and model performance was assessed by Akaike Information Criteria (AIC) and Bayesian Information Criteria (BIC). Additionally, because of differences in the mechanisms resulting in suicide based on gender and age (53–56), stratified analyses were performed. Stratification was based on gender (male, female) and age group (-39, 40–59, 60- years).

R Statistical Software v4.2.2 (57) was used for all statistical analyses. Furthermore, package lavaan v0.6-13 (58) was utilized for confirmatory factor analysis, package survey v4.1-1 (59) for analysis based on the complex sample design, and package MASS v7.3-58.2 (60) for the negative binomial regression model. All statistical tests were two-tailed, and a *P*-value of 0.05 was considered the threshold for significance. Asterisks were used to indicate the significance level for enhanced readability: * for *P* < 0.05, ** for *P* < 0.01, and *** for *P* < 0.001.

## 3 Results

### 3.1 Group comparison of regional characteristics

Demographic information and ATTS responses were collected from 1,500 participants through the 2018 KNSS. Moreover, the inclusion criteria yielded data on suicide mortality for 11,587 cases obtained via MDIS. No missing values were observed in the data.

Group comparisons were conducted by categorizing the 30 regions into those with high and low suicide rates (**Table 2**). Regions with high suicide rates exhibited older mean ages (*P* = 0.029*) and greater proportions of older adults (*P* = 0.041*). Conversely, no statistically significant differences were observed in gender or participant count. Seoul, Korea’s largest city and capital, and its metropolitan areas predominantly fell within the low suicide rate regions (**Supplementary Table 1**). Nevertheless, the regional population did not exhibit any significant group differences.

**Table 2:** Group comparison of basic regional characteristics.

	Mean (SD) of regions		Statistic	*P*
	High suicidal rate (n = 15)	Low suicidal rate (n = 15)		
Mean age	46.5 (2.9)	44.5 (1.1)	*W* = 165	0.029*
Older adults (%)	21.5 (5.6)	17.6 (2.4)	*W* = 162	0.041*
Females (%)	48.5 (3.5)	49.7 (1.8)	*W* = 73	0.11
Number of participants	48.7 (8.3)	51.3 (14.1)	*W* = 91	0.38
Regional population	1,179 K (281 K)	1,489 K (557 K)	*t* = -1.9 (*df* = 20.7)	0.07
Permissiveness	-0.170 (0.186)	0.163 (0.280)	*t* = -3.8 (*df* = 24.3)	<0.001***
Unjustified behavior	-0.022 (0.209)	-0.017 (0.172)	*t* = -0.1 (*df* = 27.0)	0.94
Readiness to help/Preventability	0.004 (0.185)	-0.007 (0.079)	*t* = 0.2 (*df* = 19.0)	0.84
Employed (%)	67.3 (10.6)	70.5 (10.7)	*W* = 85	0.27
College-educated (%)	39.3 (13.8)	51.8 (10.0)	*t* = -2.8 (*df* = 25.6)	0.009**
Religious (%)	42.3 (7.8)	39.7 (9.1)	*W* = 142	0.23
Suicidal ideation (%)	20.2 (9.8)	18.6 (13.4)	*t* = 0.4 (*df* = 25.7)	0.72

A total of 30 regions were categorized into two groups based on suicide rates. Wilcoxon’s test was used for non-normally distributed variables; otherwise, Welch’s t-test was used. SD standard deviation, *df* degree of freedom, 1 K 1000, *P < 0.05, **P < 0.01, ***P < 0.001.

Regarding factor scores, PA was higher in regions with low suicide rates (*P* < 0.001***), while UB and RP showed no group difference. Participants in regions with low suicide rates were more likely to have completed college-level education (*P* = 0.009**), but no significant differences were observed in employment and religiousness rates. The lifetime prevalence of suicidal ideation was found to be approximately 20%, with no observed group difference.

### 3.2 Regression models

**Figure 1:**
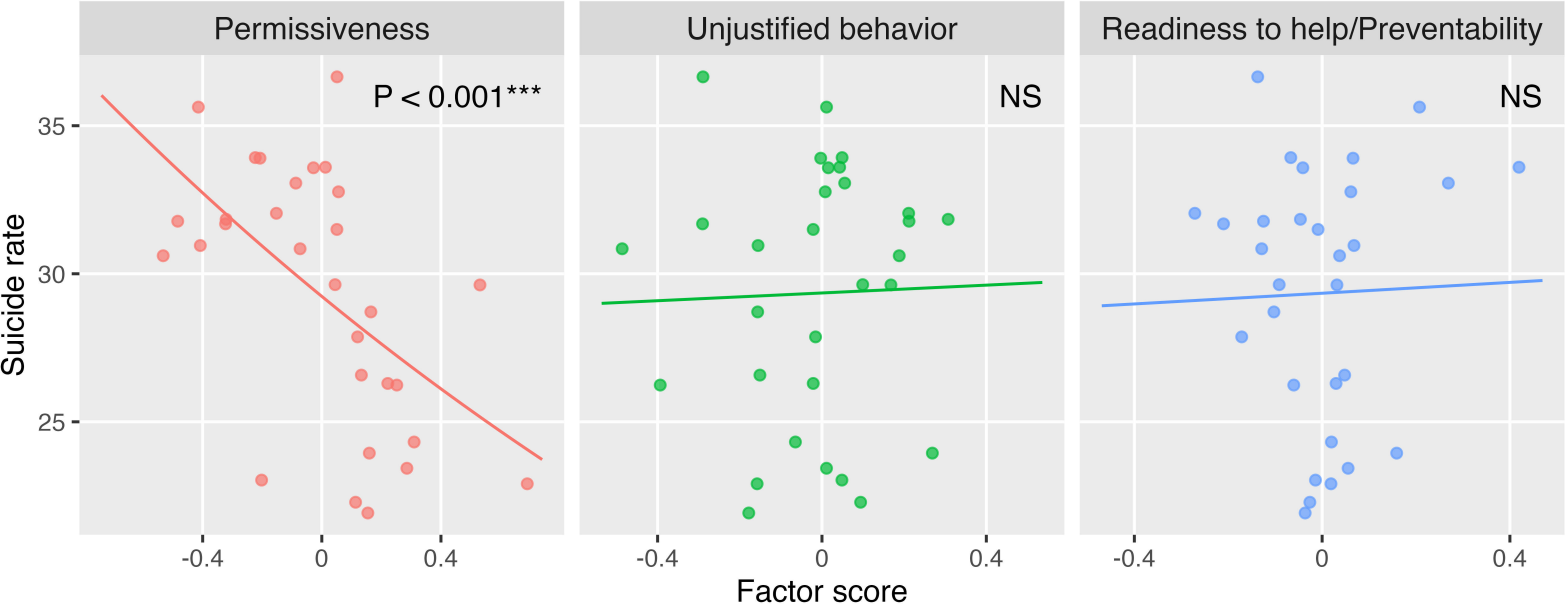
Factor scores and suicide mortality rates. Regression lines from a univariate negative binomial regression model are displayed together. Each point represents one region. A zero score indicates an average level, while positive and negative scores indicate above-average agreement and disagreement, respectively. Suicide rates were calculated per 100,000 people. *NS* not significant, *P < 0.05, **P < 0.01, ***P < 0.001.

A scatter plot of factor scores and suicide rates for each region is illustrated along with a regression line based on Model_univ_ (**Figure 1**). A significant decrease in suicide mortality was observed as the PA score increased (*P* < 0.001***). Comparing the 20^th^ to 80^th^ percentile of the “Permissiveness” score, predicted suicide rates decreased by 11.2%, equating to a difference of 34.9 cases per year in a virtual region with a population of a million. However, no significant association was identified for UB and RP.

The results of univariate and four multiple regression models were acquired (**Table 3**). PA was significantly associated with decreased suicide mortality in all the regression models. Conversely, UB was not associated with suicide in any model. RP showed no significant association with suicide in any model, except in Model 4, where the quadratic term was significantly associated. This association indicated that suicide mortality increased as the factor score deviated further from the mean. The lowest values of both AIC and BIC were observed in Model_univ_ with PA, followed by Model 3 with PA.

**Table 3:** Results of negative binomial regression models.

Factor	*IRR* (95% CI)	*Z*	*P*	*ΔAIC*	*ΔBIC*
Model_univ_					
Permissiveness	0.75 (0.64-0.89)	-3.44	<0.001***	0.00	0.00
Unjustified behavior	1.02 (0.76-1.37)	0.15	0.88	9.94	9.94
Readiness to help/Preventability	1.03 (0.70-1.52)	0.16	0.88	9.94	9.94
Model 1					
Permissiveness	0.78 (0.66-0.93)	-2.75	0.006**	2.86	5.66
Unjustified behavior	0.98 (0.74-1.29)	-0.17	0.87	9.68	12.48
Readiness to help/Preventability	1.04 (0.72-1.50)	0.21	0.83	9.66	12.46
Model 2					
Permissiveness	0.79 (0.66-0.93)	-2.73	0.006**	4.14	8.34
Unjustified behavior	1.00 (0.76-1.33)	0.01	0.99	10.88	15.09
Readiness to help/Preventability	1.01 (0.70-1.46)	0.07	0.95	10.88	15.08
Model 3					
Permissiveness	0.81 (0.69-0.95)	-2.52	0.012*	0.38	4.59
Unjustified behavior	0.93 (0.72-1.20)	-0.56	0.57	5.96	10.17
Readiness to help/Preventability	0.98 (0.70-1.38)	-0.10	0.92	6.28	10.48
Model 4					
Permissiveness: Linear	0.79 (0.66–0.94)	-2.62	0.009**	4.37	8.58
Quadratic	0.86 (0.57–1.31)	-0.70	0.49		
Unjustified behavior: Linear	1.03 (0.76–1.40)	0.20	0.84	11.06	15.26
Quadratic	1.58 (0.51–4.92)	0.79	0.43		
Readiness to help/Preventability: Linear	0.81 (0.57–1.14)	-1.20	0.23	2.67	6.87
Quadratic	9.88 (2.46–39.63)	3.23	0.001**		

Model_univ_ refers to a univariate regression model, and Model 1 is adjusted for gender and older adult proportions. Models 2 and 3 are further adjusted for suicidal ideation and college education, respectively, to Model 1. Model 4 was adjusted by adding a quadratic term of the factor score to Model 1, and both linear and quadratic terms are displayed. Information criteria are displayed in delta values, a difference from the minimum. IRR incidence rate ratio, CI confidence interval, AIC Akaike’s information criteria, BIC Bayesian information criteria, *P < 0.05, **P < 0.01, ***P < 0.001.

### 3.3 Stratified analysis

**Figure 2:**
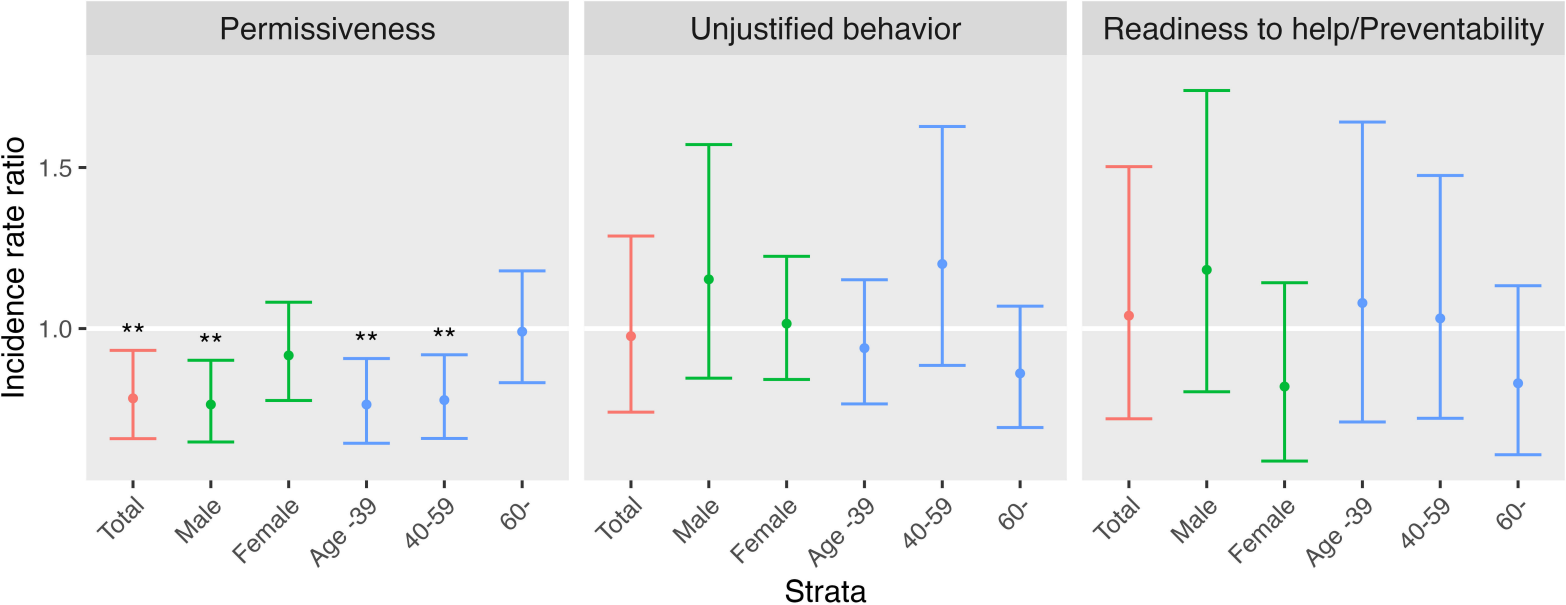
Gender and age group stratified analysis in Model 1. It displays the incidence rate ratios of suicide mortality with 95% confidence intervals indicated by error bars. *P < 0.05, **P < 0.01, ***P < 0.001.

Stratified analysis was conducted in Model 1 to examine the impact of gender and age (**Figure 2**). Results showed that PA was associated with decreased IRR of suicide in males and ages under 60 years. UB and RP did not exhibit significant associations in any strata, and no factor significantly impacted the IRR for females and older adults. Findings from other models were consistent, except for the female stratum in Model_univ_ (**Supplementary Figure 2**).

## 4 Discussion

This study investigated the effect of community attitudes on real-world suicide through an ecological methodology. Attitudes, which were aggregated by region, demonstrated various relationships with suicide mortality, also aggregated by region. Regions where suicide was permissible (as measured by PA) had a lower suicide rate. This association remained significant even after adjusting for gender, the proportion of older adults, suicidal ideation, and college education. After stratification, significant associations were still present in males, younger adults, and middle-aged adults. Oppositional attitudes toward suicide (as measured by UB) showed no significant connection to suicide rates in any stratum or model. Proactive attitudes toward suicide prevention (as measured by RP) demonstrated a significant quadratic association with suicide rates.

It is worth noting that regional variations in the proportion of individuals who have experienced suicidal behavior, although a minority, may influence the association. Additionally, Seoul and its metropolitan area, which have a high socioeconomic and education level (61), showed low suicide rates, which may confound the association. Nevertheless, the association between permissive attitudes and low suicide mortality was still significant after adjusting for either suicidal ideation or college education. Furthermore, the performance of the univariate model, as measured by AIC and BIC, was superior to that of the other multivariate models. This implies that permissive attitudes effectively and independently predict regional suicide rates, though overinterpretation should be avoided.

As mentioned earlier, previous studies have established a positive association between pro-suicidal attitudes and suicidal behavior at the individual level (21–30), including a study of the 2013 and 2018 KNSS data (45). However, this study reports a negative association between suicide rates and permissive attitudes, where permissive regions had lower suicide rates despite permissive individuals being more likely to engage in suicidal behavior. A more comprehensive perspective must consider individuals at risk of suicide and their surrounding communities to reconcile these conflicting results. In a region or community, suicide is uncommon, and most people do not intend to or attempt it (62). Therefore, attitudes aggregated through ecological analysis are more likely to reflect non-suicidal individuals’ views. Our findings, based on this context, show that if a community has permissive attitudes toward suicide, individuals at risk of suicide are less likely to die by suicide.

Reverse causality is one possible explanation, whereby if suicide is more frequent, the community may become less permissive. However, this explanation is less likely, as previous studies consistently report that losing a close person by suicide can result in a more permissive or normalizing attitude toward suicide (19, 63). Another possible explanation is that high levels of permissive attitudes may prevent suicide. Individuals’ attitudes and stigma significantly affect their behavior in disclosing their struggles, seeking help, and utilizing mental health services (64–66). As suicide occurs within socio-cultural contexts, community attitudes toward suicide may affect the behavior of suicidal individuals.

Taking community attitudes as a variable that operates in a socio-cultural context, diverse possibilities exist regarding their role in contemporary suicide theories. The theoretical explanations of 3ST and IMV on the point where social factors affect the progression of suicidal behavior differ slightly. From the perspective of 3ST (16), community attitudes can be seen as related to connectedness, which is defined as a broad construct encompassing various social elements that prevent individuals’ suicidal ideation from escalating into a strong desire. In this sense, individuals surrounded by permissive neighbors may be more likely to seek and receive help when at risk of suicide, leading to a lower suicide rate in the community. Conversely, the perspective of IMV (15) considers social interaction variables as motivational moderators that govern the eventual occurrence of suicidal ideation in vulnerable individuals. Permissive attitudes in a community may prevent defeated, humiliated, and entrapped individuals from developing suicidal ideation. Besides, the influence of contextual variables among social factors is not explicitly organized within these theoretical systems (67, 68). Therefore, an elaboration on the role of community attitudes can aid in integrating the influence of socio-cultural context into modern suicide theory, contributing to its advancement.

The stratified analysis revealed that male suicide is more strongly associated with attitudes than that in females. Previous studies have established that while male suicide is more common than female suicide, females are more likely to attempt suicide and seek help (69, 70). The effect of permissive attitudes on male suicide may be more powerful as male suicidal behavior is often silent and stealthy. In addition, older adults, who have the highest suicide rate among the Korean population, were least affected by attitudes when stratified by age group. In Korea, socioeconomic status, such as poverty, might be a more significant risk factor for suicide in older adults (51, 52), outweighing the effect of attitudes. Therefore, suicide prevention strategies for females and elderly individuals should consider other factors beyond attitudes. Attitudinal approaches to suicide prevention may be most effective in males and younger individuals.

No significant associations between oppositional attitudes toward suicide and suicide mortality were observed across models or strata. Participants in the 2018 KNSS uniformly agreed with items in the UB (**Table 1**), which may have resulted in an insignificant association due to the narrow distribution. The cognitive-emotional elements of public stigma, namely stereotypes and prejudice (71), are semantically related to oppositional attitudes. Previous research has revealed high levels of public stigma among Korean policymakers (72), with this elevated stigma closely linked to various cultural factors within the country (73). It is plausible that the distribution of oppositional attitudes serves as a manifestation of public stigma. Moreover, inconsistencies in associations between community attitudes and suicide observed in previous studies may be attributable to the heterogeneity of public stigma across time and regions. As a result, it would be inappropriate to conclude that public stigma is not a significant issue in Korean suicide merely because oppositional attitudes toward suicide have not exhibited an association with suicide mortality.

The significant quadratic association between proactive attitudes toward suicide prevention and suicide mortality suggests that proactive attitudes may have a bidirectional relationship with suicide. Regions with average levels of proactive attitudes showed lower suicide rates, while regions with either high or low attitude scores exhibited higher suicide rates. It is plausible that in regions with low proactive attitudes, weaker suicide prevention efforts lead to higher suicide mortality. However, this does not explain why regions with high proactive attitudes also have high suicide rates. Thus, it is possible that high suicide rates may, in turn, elevate proactive attitudes. In fact, since 2011, a nationwide suicide prevention education program has been implemented, and by 2019, 1.2 million people had completed the program (5). Promoting proactive attitudes is one of the primary objectives of these programs, and it is possible that the program’s implementation or efficacy was particularly strong in some regions where suicide is more prevalent.

The reliability of UB and RP warrants discussion. No consensus exists on a definitive threshold for reliability, as this depends on the study’s purpose (74). ATTS encompasses a broad spectrum of human attitudes, which are inherently non-clear-cut and ambivalent. The ATTS features a limited number of items because of the need for feasibility in large-scale surveys (25). Furthermore, KNSS, with its goal of nationwide information gathering and wide age range coverage, inevitably introduces heterogeneity into the sample. Considering these circumstances and the objectives of this study, it is reasonable for attitude factors to carry some degree of heterogeneity; thus, the reliability levels of these factors are deemed acceptable. Other studies measuring attitudes toward suicide have also reported similar levels of reliability (24–26).

### 4.1 Implications and future directions

The findings of this study bear significant implications for both the research field and practical application. First, our investigation provides theoretical evidence that the effects of community attitudes and individual attitudes toward suicide may differ. Individuals with permissive attitudes may be more likely to engage in suicidal behavior (18). Extrapolating this relationship to the group level or other contexts, however, may result in oversimplification. Future studies on attitudes toward suicide should differentiate between the attitudes of at-risk individuals and those of the surroundings.

Second, the results imply that addressing community attitudes could serve as a practical approach to suicide prevention. However, modifying attitude is a complex process, and short-term suicide prevention education has not shown a significant association with permissive attitudes toward suicide (75, 76). Furthermore, proactive attitudes toward suicide prevention are already prevalent in Korea and appear to have limited effect on suicide mortality. Consequently, alternative strategies beyond temporary measures are required, as well as short-term educational efforts focusing on public stigma and technical aspects. Community attitudes may function as surrogate markers for sociological variables that influence the phenomenon of suicide, such as individualism-collectivism (77) and social integration (12). This study might provide evidence that Korean society necessitates an extended, long-term discourse encompassing diverse values surrounding suicide and life.

To corroborate and actualize these implications, further elaboration on how community attitudes function as contextual variables is essential. Multilevel analysis suits this inquiry (78). It enables us to distinguish between group-level effects and cross-level interactions and the identification of the impact of community attitudes on the development of suicidal behavior. Unfortunately, the sample size of 1,500 participants and 30 regions included in this study was insufficient to conduct a multilevel analysis because of the low base rate of suicidal behavior in the general population, which would necessitate a larger sample size (79). Future studies with larger sample sizes, measuring help-seeking variables, and utilizing longitudinal design may aid in elucidating the relationship between attitudes and suicidal behavior more comprehensively. A better understanding of the socio-cultural context through community attitudes is required to address the stagnant suicide rates in Korea.

### 4.2 Limitations

While this study has several strengths, such as utilizing MDIS to categorize real-world suicide into specific populations and employing a factor structure supported by confirmatory factor analysis, it also presents some limitations. First, the study’s cross-sectional design precludes any meaningful discussions about causality. Causal inferences drawn from this study should be cautiously approached and require additional evidence. Second, an ecological study is subject to the ecological bias or fallacy, where the observed associations at the group level may differ from the actual associations at the individual level (80, 81). Thus, the regional-level associations observed in this study cannot be equated with individual-level associations, and we discussed the possibility that the associations between permissive attitudes and suicide mortality may differ at different levels. Third, the regression models remained simplistic because of the limited number of observations across 30 regions (82). A greater number of regional units would allow for considering various covariates and their interactions in a single model. Fourth, the factor scores from the ATTS were derived from an average of 50 participants per region, which is relatively small to ensure representativeness. This could introduce measurement errors in regional attitudes and potentially affect the study’s findings. Finally, while maintaining a relatively uniform cultural context within a single nation may be considered a strength from certain perspectives (83), it could also limit generalizability. It would be necessary to examine whether these associations can be replicated in different population and cultural contexts outside of South Korea.

## 5 Conclusion

In this study, we explored the relationship between suicide-related community attitudes and suicide mortality based on a nationwide survey and real-world suicide data in South Korea. Permissive attitudes were associated with reduced suicide mortality at the group level, with this relationship affecting males and individuals younger than 60 years more than their counterparts. It is imperative to note that attitudes may behave differently at the individual and group levels, and exercising caution when oversimplifying permissive attitudes as a risk factor is essential. Moreover, our findings show that community attitudes could play a vital role in shaping future strategies to address the gaps in Korean suicide prevention efforts. Although further studies are required, our findings might contribute to a deeper understanding of the modifiable social determinants of suicide and enhance early detection and intervention for individuals on the verge of suicide.

## Data Availability

Publicly available datasets were analyzed in this study. The 2018 Korea National Survey on Suicide data can be found at: Korean Statistical Information Service (https://kosis.kr/). Microdata on suicide mortality is available upon appropriate request from: MicroData Integrated System (https://mdis.kostat.go.kr/)
