# Supplementary material for: Effect of community attitudes on suicide mortality in South Korea: a nationwide ecological study
Source: Front Psychiatry. 2024 Sep 16;15:1423609. doi: 10.3389/fpsyt.2024.1423609 (PMC11439762; doi:10.3389/fpsyt.2024.1423609)
Supplement: Supplementary file 1 [file DataSheet1.docx]

**Supplementary materials**

Supplementary Fig. 1 Determination of the number of factors. The eigenvalue (top graph) decreased below 1 after three factors, while parallel analysis suggested more than 10 factors. The very simple structure test (middle graph) did not produce a single prominent peak, although values for two to three factors were comparatively higher. The minimal average partial correlation test (bottom graph) exhibited its lowest point at three factors. Based on these findings, it is reasonable to determine the number of factors as three.

Supplementary Fig. 2 Path diagram of the confirmatory factor analysis. *df* degree of freedom, *SRMR* standardized root mean square residual, *RMSEA* root mean square error of approximation, *CFI* comparative fit index.

| **Region** | **Number of**  **participants** | **Mean age** | **Older adults** | **Female** | **Suicide rate** |
| --- | --- | --- | --- | --- | --- |
| Chungnam | 60 | 46.1 | 19.5% | 47.4% | 36.7 |
| Jeju | 40 | 45.2 | 16.7% | 48.5% | 35.6 |
| Gangwon | 50 | 46.7 | 21.7% | 48.3% | 33.9 |
| Gyeongbuk-South | 40 | 44.0 | 18.5% | 49.3% | 33.9 |
| Gyeongnam-East | 50 | 43.5 | 19.9% | 55.8% | 33.6 |
| Jeonbuk | 60 | 45.7 | 21.7% | 48.9% | 33.6 |
| Chungbuk | 50 | 44.0 | 19.1% | 48.1% | 33.1 |
| Gyeongnam-West | 50 | 51.7 | 31.5% | 39.1% | 32.8 |
| Incheon-South | 41 | 45.4 | 15.6% | 51.0% | 32.0 |
| Jeonnam | 60 | 47.4 | 22.3% | 48.0% | 31.8 |
| Gyeongbuk-North | 40 | 52.0 | 31.8% | 47.0% | 31.8 |
| Ulsan | 40 | 44.0 | 15.2% | 47.5% | 31.7 |
| Busan-West | 60 | 47.9 | 23.0% | 52.5% | 31.5 |
| Busan-East | 40 | 50.5 | 30.7% | 47.4% | 31.0 |
| Daejeon | 50 | 43.9 | 15.9% | 49.4% | 30.8 |
| Daegu | 70 | 47.3 | 23.0% | 50.0% | 30.6 |
| Incheon-North | 40 | 43.3 | 18.3% | 47.3% | 29.6 |
| Gyeonggi-Middle2 | 70 | 44.1 | 16.0% | 50.5% | 29.6 |
| Gyeonggi-North | 60 | 45.9 | 18.3% | 47.8% | 28.7 |
| Gwangju | 40 | 44.8 | 18.6% | 49.9% | 27.9 |
| Gyeonggi-South2 | 49 | 43.9 | 14.8% | 50.4% | 26.6 |
| Seoul-Northeast1 | 40 | 44.8 | 20.4% | 50.4% | 26.3 |
| Sejong | 30 | 44.7 | 16.7% | 49.0% | 26.2 |
| Seoul-Northeast2 | 40 | 44.4 | 18.2% | 50.6% | 24.3 |
| Gyeonggi-South2 | 70 | 43.5 | 12.8% | 45.4% | 23.9 |
| Seoul-Southwest1 | 40 | 44.3 | 18.2% | 50.6% | 23.4 |
| Seoul-Southwest2 | 40 | 43.3 | 16.0% | 50.4% | 23.0 |
| Seoul-Southeast | 60 | 43.8 | 16.8% | 52.0% | 22.9 |
| Seoul-Northwest | 50 | 45.0 | 18.2% | 52.2% | 22.3 |
| Gyeonggi-Middle1 | 70 | 45.1 | 18.2% | 49.7% | 21.9 |

Supplementary Table 1. Regional demographic characteristics and suicide rates. Regions are displayed in descending order of suicide rate per 100,000 people.


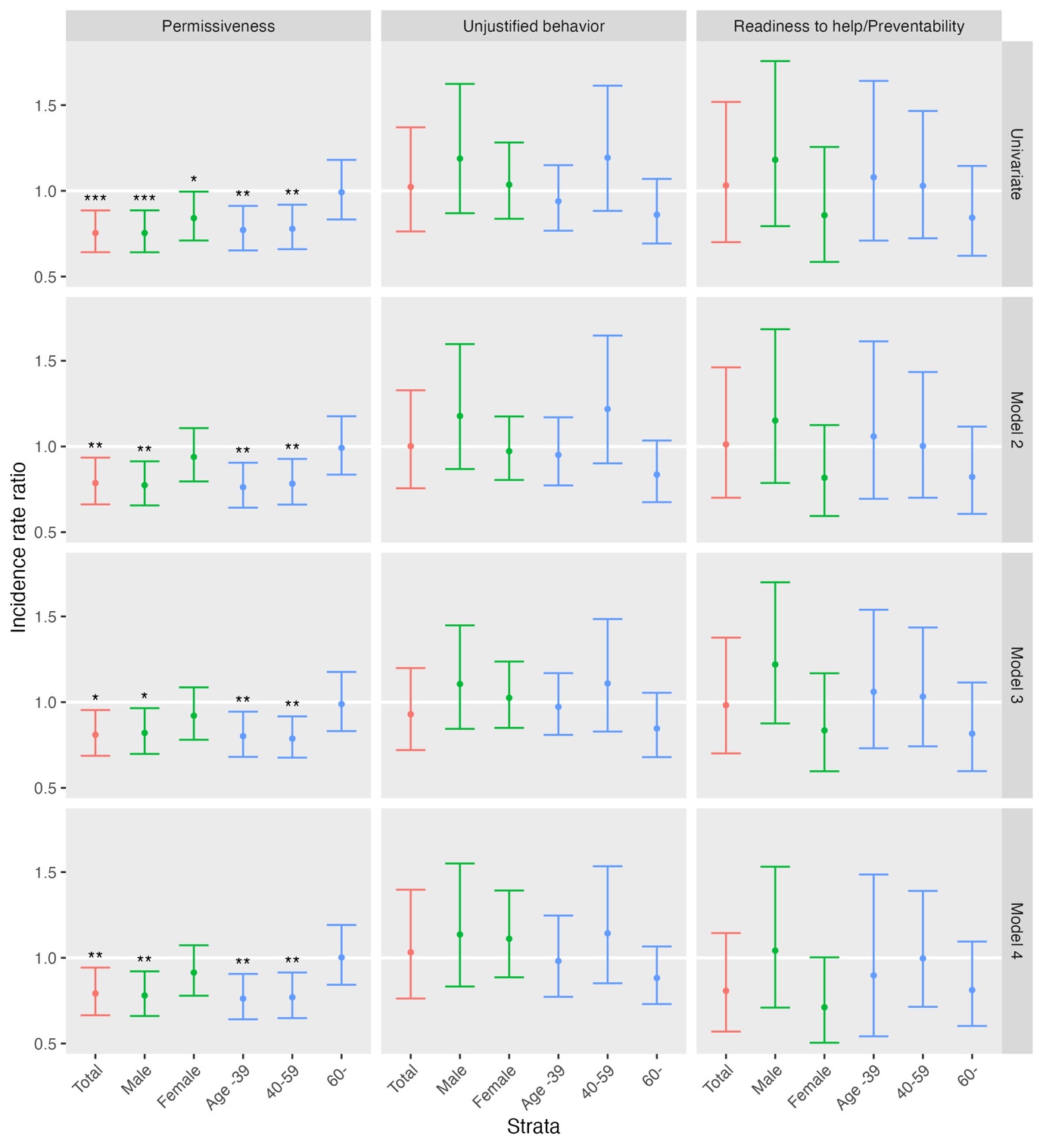


Supplementary Fig. 3 Stratified analysis replicated in univariate and multivariate models. (Result of Model 1 can be found in Fig 2).
